# Xanthine oxidase activity in patients with age-related cataract associated with hypertension

**DOI:** 10.1590/1414-431X20176129

**Published:** 2018-03-26

**Authors:** B. Kisic, D. Miric, L. Zoric, J.V. Rasic, R. Grbic, Lj.M. Popovic, A.M. Arsic

**Affiliations:** 1 Institute of Biochemistry, Faculty of Medicine, University of Pristina, Kosovska Mitrovica, Serbia; 2 Clinic for Eye Diseases, Faculty of Medicine, University of Pristina, Kosovska Mitrovica, Serbia; 3 Institute of Pharmacology, Faculty of Medicine, University of Pristina, Kosovska Mitrovica, Serbia; 4 Clinic for General and Orthopedics Surgery, Faculty of Medicine, University of Pristina, Kosovska Mitrovica, Serbia; 5 Institute of Pathophysiology, Faculty of Medicine, University of Pristina, Kosovska Mitrovica, Serbia; 6 Department for Laboratory Investigation, Railway Health Care Institute, Novi Sad, Serbia

**Keywords:** Age-related cataract, Hypertension, Xanthine oxidase, Myeloperoxidase

## Abstract

Reactive oxygen species and lipid peroxidation are important factors that contribute to the development of age-related cataract. The study included 130 patients with age-related cataract, 69 of whom were diagnosed with hypertension (HT), 20 with hypertension and type 2 diabetes mellitus (DM), and 41 had no accompanying condition. The following parameters were measured in the serum of the examinees: products of lipid peroxidation malondialdehyde (MDA) and lipofuscin-like fluorophores (LLF), activity of prooxidative enzymes xanthine oxidase (XO) and myeloperoxidase (MPO), antioxidant enzymes superoxide dismutase (SOD) and glutathione peroxidase (GPx), the concentration of thiol groups, and the ferric reducing activity of plasma. The activity of prooxidative enzymes XO and MPO was higher in the plasma of patients with HT (XO=9.0±1.2 U/L; MPO=77.3±8.4 U/L) and with HT and DM (XO=11.9±0.9 U/L; MPO=89.5±5.0 U/L) compared to patients with age-related cataract (XO=6.2±0.9 U/L; MPO=52.4±6.3 U/L; P<0.01). Our research has shown that patients with age-related cataract and hypertension were exposed to increased oxidative damage of biomolecules, based on the increased plasma LLF and MDA content and decreased levels of thiol groups. Oxidative changes of biomolecules in these patients were associated with increased activity of the XO, MPO, and GPx enzymes and a lower extracellular SOD activity and total ferric reductive ability of plasma.

## Introduction

Oxidative modifications of various biomolecules constantly occur in tissues throughout an individual's life, but in patients with age-related cataract (ARC), oxidative modifications of proteins and lipids are obvious and a dominant metabolic feature and pathological substrate of disease (1). The development of ARC involves multiple though inter-related risk factors, including ageing process, exposure to environmental and intrinsic oxidants, diet and lifestyle factors, as well as several systemic disorders, such as diabetes mellitus (DM) and hypertension (HT) (2,3).

The origin of HT is also multifactorial, and currently not all underlying molecular mechanisms are known. Still, numerous studies indicate that reactive oxygen species (ROS) are implicated in vascular redox signaling, regulation of vascular tone, and remodeling of blood vessel walls. Conversely, increased presence of oxidants resulting in oxidative stress probably contributes to endothelial dysfunction and the development of hypertension (4). In endothelial cells, redox state is primarily regulated by a dynamic balance between endogenously synthesized nitric oxide (NO) and superoxide anion radical (O_2_^•−^), which is the main ROS within vasculature. NO is a potent vasodilator substance and its diminished availability upon reaction with O_2_^•−^ causes increased resistance of arterioles, eventually leading to the development of hypertension (5).

Although there could be several sources of oxidants in the vasculature implicated in hypertension, xanthine oxidase (XO) has received special attention in past years. In physiological conditions, this xanthine-degrading enzyme exists as a dehydrogenase, utilizing NAD^+^ as an electron acceptor in the course of conversion of hypoxanthine and xanthine to uric acid. However, limited proteolysis, oxidation of critical thiol-groups, or hypoxic conditions can induce the conversion of the enzyme to the oxidase form (6). Both xanthine dehydrogenase and XO utilize xanthine and hypoxanthine as substrates to produce uric acid and ROS.

It has been previously demonstrated that XO-derived oxidants have a strong impact on arteriolar tone and blood pressure in spontaneously hypertensive animals (7), and that XO activity represented a significant risk factor of hypertension in normotensive younger adults (8). Given that hypertension is a significant risk factor of ARC development, and that we previously demonstrated involvement of XO in oxidative damage and earlier development of ARC among diabetic subjects (9), our current study aimed to investigate the relationship between XO and hypertension in patients with ARC.

## Patients and Methods

This study included 130 patients with ARC, scheduled for cataract surgery in the Department for Ophthalmology, Health Center Kosovska Mitrovica, Serbia. Of all included patients, 41 had ARC only, 69 had ARC and hypertension, and 20 patients had ARC, HT, and type 2 DM. The study protocol was approved by the Ethics Committee of the Medical Faculty in accordance with the Helsinki Declaration. Informed consent was obtained from all individual participants included in the study. Patients with hypertension were identified by blood pressure of more than 140/90 mmHg. Their blood pressure was recorded by an auscultatory method using a sphygmomanometer.

Venous blood was collected after an overnight fasting into tubes containing solid dipotassium EDTA or into plain tubes. The tubes were centrifuged at 1351 *g* for 10 min at 4°C, serum or plasma samples were separated, and aliquoted samples were immediately stored at –80°C. All chemicals used in this study were of analytical grade.

Serum lipid profile, total cholesterol (TC), high-density lipoprotein cholesterol (HDL-C), and triglycerides (TAG) were measured using commercial enzymatic kits (Roche/Boehringer, Germany) by automated analyzer (Hitachi Instruments, Japan). Low-density lipoprotein (LDL)-cholesterol was derived according to the Friedewald's equation. Concentration of glycated hemoglobin A1C (HbA1C) was assessed by turbidimetric inhibition immunoassay. Body mass index (BMI) was calculated as the ratio between body weight in kg and the square of body height in m^2^.

The concentration of malondialdehyde (MDA), which is a relatively stable lipid peroxidation marker, was measured by a method according to Ledwozyw et al. (10) on a Safas UV/VIS spectrophotometer (Safas 2, Monaco). Absorbance of the complex produced in the reaction of MDA with 2-thiobarbituric acid (2-thiobarbituric acid, 70 mM; NaOH, 50 mM) was measured at λ=532 nm. The lipofuscin-like fluorophores (LLF) were measured at 360/430 nm (excitation/emission) in a fluorescent photometer equipped with a xenon lamp (Jenway 6285, Barloworld Scientific Ltd, UK), after delipidation of plasma with ethanol/ether (3/1, v/v) (11).

XO activity was measured by spectrophotometry based on the change in absorbance at 290 nm, at the conversion of xanthine to uric acid (xanthine, 3 mM; in 0.1 M NaOH/glycine buffer) (12). Plasma myeloperoxidase (MPO) activity was assayed in the system of 4-aminoantipyrine and phenol with hydrogen peroxide (1.7 mM) as the substrate, by monitoring the production of quinoneimine at λ=505 nm, as described by Metcalf et al. (13). One unit of MPO activity was defined as the amount of enzyme degrading 1 µmol of hydrogen peroxide per minute at 25°C.

Activity of SOD was measured by the method of Misra and Fridovich (14) based on the ability of SOD to inhibit auto-oxidation of adrenaline, with adrenaline bitartarate (7 mM) as a substrate, at alkaline pH (pH 10.2). The increase in absorbance of adrenochrome was monitored at 25°C for 3 min at λ=480 nm. One unit of SOD activity was defined as the amount of enzyme that inhibits auto-oxidation of 5 mmol adrenaline by 50%. Plasma GPx activity was measured using cumene hydroperoxide as a substrate, the method of Chin et al. (15). The absorbance of the reaction product was measured at 412 nm.

The concentration of serum -SH groups was measured with Ellman's reagent (DTNB) (16). Protein -SH groups reduced DTNB [5,5′-dithiobis-(2-nitrobenzoic acid)] producing a yellow-colored anion of 5-thio-2-nitrobenzoic acid (TNB^–^). The absorbance was read at 412 nm on the Safas UV/VIS spectrophotometer. The ferric reducing/antioxidant power assay (FRAP) is a colorimetric method for testing the ability of ferric reductive plasma, where at low pH, Fe^+3^-TPTZ reduces to Fe^+2^-TPTZ giving a blue color with maximum absorbance at 593 nm (17).

### Statistical analysis

Descriptive statistical methods, methods for testing statistical hypotheses, and methods for testing dependence were used for the analysis of primary data. Descriptive statistical methods used were the following: measure of central tendency (arithmetic mean), measure of variability (standard deviation), and relative numbers. The methods used for testing statistical hypotheses were the *t*-test for two independent samples and the single-factor ANOVA with the *post hoc* Tukey's test. The method used for dependence analysis was the Pearson linear correlation coefficient. Hypotheses were tested at the level of statistical significance (alpha level) of 0.01 and 0.05. The software program SPSS Statistics 22 (USA) was used for statistical analysis of the results.

## Results

Demographic and clinical characteristics and biochemical indicators of lipids and glycemic status in the examined patients are presented in Table 1.

**Table 1.** Basic demographical, clinical, and biochemical characteristics of patients.

	ARC	ARC and HT	ARC, HT, and DM	P value
Number of subjects, n (%)	41 (32%)	69 (53%)	20 (15%)	
Gender, n (%)				0.864
Male	21 (17)	39 (30)	9 (7)	
Female	20 (15)	30 (23)	11 (8)	
Age (years)	71.4±7.3	71.3±6.0	72.1±6.9	0.893
Mean SBP (mmHg)	120±10	152±18	154±13	<0.01
Mean DBP (mmHg)	75±5	95.±10	97±11	<0.01
Body mass index (kg/m^2^)	23.9±2.5	24.5±2.8	25.2±2.5	0.083
Baseline glucose (mmol/L)	5.4±1.3	5.5±1.5	7.9±1.9	<0.01
HbA1C (% of hemoglobin)	5.3±1.8	5.6±1.5	9.1±2.4	<0.01
Total cholesterol (mmol/L)	4.7±0.3	6.8±0.4	7.2±0.5	<0.01
HDL-cholesterol (mmol/L)	1.6±0.3	0.9±0.2	0.9±0.4	<0.01
LDL-cholesterol (mmol/L)	3.0±0.5	6.0±0.5	6.3±0.8	<0.01
Triacylglycerols (mmol/L)	1.9±0.5	2.4±0.5	2.7±0.4	<0.01

Data are reported as means±SD or number and percentage. ARC: age-related cataract; HT: hypertension; DM: diabetes mellitus; HbA1C: glycated hemoglobin A; SBP: systolic blood pressure; DBP: diastolic blood pressure; LDL: low-density lipoprotein; HDL: high-density lipoprotein. Statistical analysis was done with ANOVA the *post hoc* Tukey's test.

Table 2 shows the indicators of oxidative stress in serum of examined patients, markers of lipid peroxidation MDA and LLF, the activity of the pro-oxidative enzymes XO and MPO, the activity of antioxidant enzymes SOD and GPx, the concentration of -SH groups, and FRAP.

**Table 2.** Markers of lipid peroxidation and antioxidant enzyme activities in the blood of patients.

	ARC	ARC and HT	ARC, HT, and DM	P value
Number of subjects, n (%)	41 (32%)	69 (53.0%)	20 (15%)	
XO (U/L)	6.2±0.9	9.4±1.3	11.9±0.9	<0.01
MPO (U/L)	52.4±6.3	77.3±8.4	89.5±5.0	<0.01
LLF (RFU/mL)	48.4±5.2	79.7±11.0	92.3±4.5	<0.01
MDA (µmol/L)	4.6±0.8	6.6±0.5	8.0±0.6	<0.01
SH groups (µmol/L)	501.4±45.6	400.6±34.6	312.7±40.6	<0.01
FRAP (µmol/L)	774.2±100.1	613.9±43.9	575.9±67.6	<0.01
GPx (U/L)	185.2±12.5	235.2±19.7	287.7±11.9	<0.01
SOD (U/L)	38.7±1.2	29.9±3.0	24.1±2.7	<0.01

Data are reported as means±SD. ARC: age-related cataract; HT: hypertension; DM: diabetes mellitus; XO: xanthine oxidase; MPO: myeloperoxidase; LLF: lipofuscin-like fluorophores; MDA: malondialdehyde; SH: thiol; FRAP: ferric reducing/antioxidant power assay; GPx: glutathione peroxidase; SOD: superoxide dismutase. Statistical analysis was done with ANOVA the *post hoc* Tukey's test.

The activity of oxidant enzymes XO (6.2±0.9 U/L; P<0.01) and MPO (52.4±6.3 U/L; P<0.01) in the serum of patients with age-related cataract was significantly lower compared to patients with age-related cataract and HT (XO=9.0±1.2 U/L; MPO=77.3±8.4 U/L) and patients with HT and DM (XO=11.9±0.9 U/L; MPO=89.5±5.0 U/L). Also, there was a significant difference between the activities of both oxidant enzymes in the serum of patients with a HT and patients with HT and DM (P<0.01).

Significantly lower activity of antioxidant enzymes SOD and GPx was measured in patients with HT and DM compared to patients with only ARC.

The plasma of patients diagnosed with HT and DM in addition to ARC showed the lowest ferric reductive ability (575.9±67.6 μmol/L) compared to the patients with ARC and HT (613.9±43.9 μmol/L) and those with age-related cataract (774.2±100.1 μmol/L; P<0.01).

The highest concentration of sulfhydryl groups was measured in the plasma of normotensive ARC patients (501.4±45.6 μmol/L) compared to patients with HT (400.6±34.6 μmol/L) and those with HT and DM (312.7±40.6 μmol/L; P<0.01).

There was a significant positive correlation between plasma concentration of lipofuscin-like fluorophores (RFU/mL) and GPx activity (U/L) (n=130, r=0.750, P<0.01), between the concentration of LLF (RFU/mL) and XO activity (U/L; n=130, r=0.793, P<0.01), and between the concentration of MDA (µmol/L) and GPx activity (U/L; n=130, r=0.789, P<0.01).

**Figure 1.**
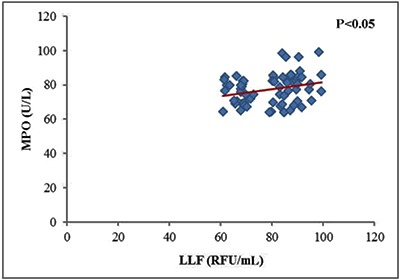
Correlation between lipofuscin-like fluorophores (LLF) and myeloperoxidase (MPO) in serum of patients with hypertension and age-related cataract (n=69, r=0.255, P<0.05).

**Figure 2.**
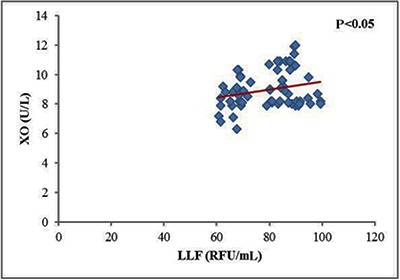
Correlation between lipofuscin-like fluorophores (LLF) and xanthine oxidase (XO) in serum of patients with hypertension and age-related cataract (n=69, r=0.244, P<0.05).

**Figure 3.**
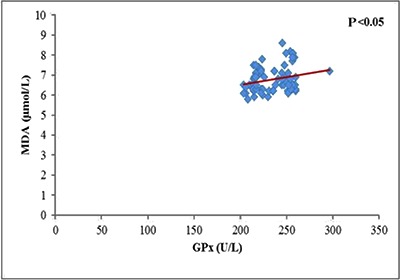
Correlation between malondialdehyde (MDA) and glutathione peroxidase (GPx) activity in serum of patients with hypertension and age-related cataract (n=69, r=0.250, P<0.05).

A significant correlation between the LLF and MPO (U/L; n=69, r=0.255, P<0.05; Figure 1), between LLF and XO (U/L; n=69, r=0.244, P<0.05; Figure 2), and between MDA (µmol/L) and GPx (U/L; n=69, r=0.250, P<0.05; Figure 3) was also found in serum of patients with hypertension. In the group of patients with ARC, HT, and DM, there was a positive correlation (r=0.459, P<0.05) between the activity of GPx (U/L) and MDA (µmol/L).

## Discussion

It is well known that, at low physiological levels, ROS play important roles in maintenance of vascular tone, and that endothelium-derived NO, synthesized by constitutively expressed NOx, acts as the major vasodilator substance. On the other hand, increased formation or impaired elimination of ROS, and consequent oxidative stress have been implicated in vascular wall dysfunction and hypertension (4,18,19) as well as in physiological ageing and in age-related diseases, including ARC (1). Moreover, population-based studies conducted in the past decade indicate that hypertension can increase the risk of ARC development (3).

Although the exact mechanism(s) of how hypertension enhances cataractogenesis is yet to be established, constantly increased blood pressure is often associated with systemic inflammation, dysregulation of glycemic control, dys- and hyperlipidemia, obesity, and oxidative stress (20). Accordingly, biomarkers of lipid oxidative damage, such as MDA and LLF, were significantly increased in hypertensive compared to normotensive ARC patients, especially in patients with co-existing diabetes. These results are consistent with a previously reported effect of hypertension on serum MDA levels (19), and with experimental evidence that systemic hypertension can modulate electrolyte homeostasis and increase MDA at both systemic levels and within lenses (21).

In the study of Khan et al., enzymatic as well as non-enzymatic antioxidants were depleted in hypertension (21), while in our study GPx activity was increased, especially in patients with co-existing diabetes. Our results were similar to Sreeja et al. (22), who reported significantly higher lens GPx activity in hypertensive compared to normotensive ARC patients. Literature data regarding effects of blood pressure on GPx activity in non-cataract subjects are also inconsistent, and erythrocyte enzyme activity was found unchanged (23) and even decreased (19), compared to normotensive controls. Although erythrocyte and plasma GPx are distinctive isoforms and thus might be differentially regulated, our results suggest that higher plasma GPx activity in hypertensive ARC could be an adaptive response to chronically increased systemic oxidative stress. Moreover, the significant correlation between GPx and MDA levels in HT and in HT and diabetic ARC group in our study further corroborate our assumption. In addition, decreased total -SH groups, FRAP, and SOD argue for intensive systemic oxidative stress in hypertensive ARC patients.

We have previously shown that increased XO expression may intensify oxidative stress at both lens and systemic levels, probably contributing to earlier development of ARC in diabetic subjects (9). Our current results further extend this and observations of others (24,25) that increased serum XO activity is significantly associated with higher degree of oxidative damage and the development of microvascular and macrovascular complications in diabetics. As presented, compared to the normotensive ARC group, patients in the hypertensive ARC group had significantly increased XO activity, and an additional enhancement of XO activity was recorded in diabetic and hypertensive ARC patients. In addition, serum XO was higher in hypertensive and diabetic ARC patients, compared to hypertensive ARC patients without diabetes.

Human endothelial cells contain cytoplasmic and membrane-bound XO, capable of producing O_2_^•−^ and H_2_O_2_, depending on whether the substrate is xanthine or hypoxanthine. In circulation, XO can also produce ROS, thereby imposing injury to vascular endothelial and other cells in close proximity, for example adhered neutrophils. The reaction between O_2_^•−^ and NO yields peroxynitrite, which is a powerful oxidant involved in nitration of tyrosine residues in proteins, also reported in hypertension and cardiovascular diseases (4). In addition, this reaction deprives local and systemic levels of vasodilator NO leading to endothelial dysfunction (20). Moreover, previous studies have shown that systemic XO activity was closely correlated to mean arterial pressure (26), and that inhibition of XO can normalize blood pressure in adolescents diagnosed with hypertension (27).

Increased XO activity was probably affected by the presence of hyperlipidemia and hypercholesterolemia in our study. In fact, the ARC with hypertension group had significantly higher concentrations of triglycerides, total cholesterol, and LDL-cholesterol levels than those with ARC only. Hypercholesterolemia is often associated with local hypoxia of the blood vessel walls, favoring the conversion of endothelial xanthine dehydrogenase to XO form. Also, hyperlipidemia and hypercholesterolemia can alter the function and/or expression of glycosaminoglycan XO receptors thereby enhancing accumulation of XO within the vascular wall. A study by Ohara et al. showed that blood vessels of cholesterol-rich diet fed animals produced three times more O_2_^•−^ than normal (28), while in another study, XO inhibitors improved endothelial function in hypercholesterolemic rabbits (29).

Previous studies have demonstrated that XO can also act as a modulator of innate immune response through induction of pro-inflammatory cytokine synthesis and activation of neutrophils and macrophages, contributing to atherosclerosis development (30). Upon activation, neutrophils release some of their MPO from azurophilic granules into the blood. MPO is a pro-oxidant enzyme that catalyzes oxidation of chlorine ions by hydrogen peroxide to hypochlorite, which is a strong oxidizing agent. MPO-derived oxidants present in the vasculature can further react with NO thereby reducing its availability, and probably contributing to vascular dysfunction and hypertension (31). Accordingly, serum MPO activity was significantly higher in hypertensive ARC patients in the current study. It can be assumed that increased activity of XO in hypertension and consequently greater production of O_2_^•−^ could have some impact on activation of phagocytes and the release of MPO into extracellular space. It was previously demonstrated that O_2_^•−^ has an important role in regulation of MPO catalytic cycles, as it allows uninterrupted development of the chlorinating MPO cycle, thus intensifying oxidative damage (32). MPO-derived oxidants can damage the vascular wall constituents and reduce the elasticity. Also, MPO-derived oxidants can initiate lipid peroxidation as they readily react with polyunsaturated fatty acids generating oxidized fluorescent products (33). Accordingly, there was a significant positive correlation between the level of LLF and MPO activity, which probably reflects a prolonged pro-oxidant activity of MPO in patients with hypertension.

Results of our current study indicated that hypertension was associated with increased systemic activity of pro-oxidant enzymes XO and MPO in patients with ARC. We have also shown that endogenous antioxidant defense mechanisms were dysregulated in ARC patients with hypertension, as plasma GPx was upregulated, while the other antioxidants, including total thiol groups, SOD and FRAP, were significantly reduced. These results indicate that hypertension promoted oxidative stress in ARC patients. In such conditions, the supply of antioxidants and vitamins to eye tissues may be insufficient to protect the eye lens from further oxidative modifications, thus contributing to the development of ARC.
